# Prenatal care in Southern Brazil: coverage, trend and disparities

**DOI:** 10.11606/S1518-8787.2019053000968

**Published:** 2019-04-26

**Authors:** Janaina S Saavedra, Juraci A Cesar, Angélica O Linhares

**Affiliations:** I Universidade Federal do Rio Grande. Faculdade de Medicina. Programa de Pós-Graduação em Ciências da Saúde. Rio Grande, RS, Brasil; II Universidade Federal do Rio Grande. Faculdade de Medicina. Programa de Pós-Graduação em Saúde Pública. Rio Grande, RS, Brasil; III Universidade Federal de Pelotas. Faculdade de Nutrição. Programa de Pós-Graduação em Nutrição e Alimentos. Pelotas, RS, Brasil

**Keywords:** Prenatal Care, utilization, Health Services Coverage, Coverage Equity, Health Status Disparities, Health Evaluation, Cuidado Pré-Natal, utilização, Cobertura de Serviços de Saúde, Equidade em Cobertura, Disparidades nos Níveis de Saúde, Avaliação em Saúde

## Abstract

**OBJECTIVE:**

To estimate coverage, examine trend and assess the disparity reduction regarding household income during prenatal care between mothers living in Rio Grande, state of Rio Grande do Sul, in 2007, 2010, 2013 and 2016.

**METHODS:**

This study included all recent mothers living in this municipality, between 1/1 and 12/31 of those years, who had a child weighing more than 500 grams or 20 weeks of gestational age in one of the only two local maternity hospitals. Trained interviewers applied, still in the hospital and up to 48 hours after delivery, a unique and standardized questionnaire, seeking to investigate maternal demographic and reproductive characteristics, the socioeconomic conditions of the family and the assistance received during pregnancy and childbirth. To assess the adequacy of prenatal care, the criteria proposed by Takeda were used, which considers only the number of prenatal appointments and gestational age at initiation, and by Silveira et al., who in addition to these two variables, considers the achievement of some laboratory tests. Chi-square tests were used to compare proportions and assess the linear trend.

**RESULTS:**

The total of 10,669 recent mothers were included in this survey (96.8% of the total). Prenatal coverage substantially increased between 2007 and 2016. According to Takeda, it rose from 69% to 80%, while for Silveira et al., it increased from 21% to 55%. This improvement occurred for all income groups (p < 0.01). The disparity between the extreme categories of income reduced, according to Takeda, and increased according to Silveira et al.

**CONCLUSIONS:**

The provision of prenatal care, considering only the number of appointments and the early start, occurred in greater proportion among the poorest. However, only the richest recent mothers were contemplated with more elaborate care, such as laboratory tests, which increased the disparities in the provision of prenatal care.

## RESUMO

**OBJETIVO:**

Estimar a cobertura, examinar a tendência e avaliar se houve redução da disparidade em relação à renda familiar na realização de pré-natal adequado entre puérperas residentes em Rio Grande, RS, nos anos de 2007, 2010, 2013 e 2016.

**MÉTODOS:**

Foram incluídas neste estudo todas as puérperas residentes nesse município que, entre 1/1 a 31/12 desses anos, tiveram filho com peso superior a 500 gramas ou 20 semanas de idade gestacional em alguma das duas únicas maternidades locais. Entrevistadoras treinadas aplicaram, ainda no hospital e em até 48 horas após o parto, questionário único e padronizado, buscando investigar as características demográficas e reprodutivas maternas, as condições socioeconômicas da família e a assistência recebida durante a gestação e parto. Para avaliação da adequação do pré-natal, foram utilizados os critérios propostos por Takeda, que considera apenas o número de consultas pré-natais e a idade gestacional de início, e de Silveira et al., que além dessas duas variáveis, leva em conta a realização de alguns testes laboratoriais. Foram utilizados os testes qui-quadrado para comparar proporções e avaliar tendência linear.

**RESULTADOS:**

Foram incluídas neste inquérito 10.669 puérperas (96,8% do total). Verificou-se substancial aumento na cobertura de pré-natal adequado entre 2007 e 2016. Segundo Takeda, passou de 69% para 80%, enquanto para Silveira et al. aumentou de 21% para 55%. Essa melhora no período ocorreu para todos os grupos de renda (p < 0,01). Houve redução na disparidade entre as categorias extremas de renda segundo Takeda e aumento acentuado segundo Silveira et al.

**CONCLUSÕES:**

A oferta de pré-natal, considerando apenas o número de consultas e o início precoce, ocorreu em maior proporção entre as mais pobres. No entanto, ao oferecer cuidados mais elaborados, como exames laboratoriais, estes alcançaram principalmente as puérperas mais ricas, aumentando assim as disparidades na oferta da assistência pré-natal.

## INTRODUCTION

Prenatal care reduces the occurrence of intrauterine growth restriction, low birth weight, prematurity, neonatal mortality and malnutrition in children^1^ ^,^ ^2^. The better the quality of the services offered, the greater the impact on maternal and child health and the lower the morbidity and mortality rate in this population^1^ ^,^ ^3^.

This quality has been measured by the combination of care offered to pregnant women, including, in general, number of appointments, gestational age at initiation of the prenatal care and, more recently, laboratory tests. As a rule, three categories were originated: adequate, intermediate and inadequate^4–6^.

Several studies have reported the existence of inequality in prenatal care^7–11^. Most of them have done this after the identification of associated factors and found that pregnant women with the worst socioeconomic status are more likely to receive inadequate care^5–10^. Other studies have made more sophisticated analyses and shown that the inequality is decreasing because the proportion of pregnant women beginning prenatal care is increasing, and the number of appointments made by them approaches, more and more, those with a better socioeconomic level. The reduction in the difference between the rich and the poor suggests that the inequality in prenatal care has been decreasing^11^ ^,^ ^12^.

Perinatal studies conducted every three years in the municipality of Rio Grande, state of Rio Grande do Sul, since 2007, have been collecting information about the care received by pregnant women since the beginning of prenatal until the immediate postpartum. These data allow the completion of this assessment in a period of 10 years (2007–2016).

This study has three objectives: 1) estimate the adequate prenatal coverage according to the criteria proposed by Takeda and Silveira et al. between 2007 and 2016 in this municipality, 2) meet the trend of this adequacy and 3) verify how this coverage behaved during this period according to the household income.

## METHODS

The municipality of Rio Grande is located in the coastal stretch of the south of Rio Grande do Sul state, 300 km from Porto Alegre. Between 2007 and 2016, the data collection period, its population increased from 197 thousand to about 213 thousand inhabitants. Throughout this period, the gross domestic product (GDP) increased from R$19.6 to R$41.3 thousand^13^, while the employment relationship doubled, going from 52,875 to 107.338^14^. The infant mortality coefficient remained around 11.5/1,000^15^, while little changed in the public health system, except for the Family Health Strategy (FHS), which went from 10 to 36 teams over the period. The same services remained in the others: 32 basic health units, three outpatient clinics and two hospitals, the University Hospital of the Universidade Federal do Rio Grande being destined entirely to the Unified Health System.

This research is part of the Perinatal Studies of Rio Grande, conducted in the municipality every three years since 2007 to assess the care received by mothers from the beginning of pregnancy until immediate postpartum. Data presented here have a time series that included all births that occurred between 1/1 and 12/31 2007, 2010, 2013 and 2016 in only two local maternity hospitals. To be included in the study, mothers should reside in the rural or urban area of the municipality of Rio Grande, and their children should have at least 500 grams or 20 weeks of gestational age at birth.

The sample size was estimated a posteriori based on the prevalence of 81.3 for Takeda^4^ in 2016 and 43.8 for Silveira et al.^5^ in 2010. Thus, from the “n” available (10,331), for a 95% confidence level, it would be possible to work with a margin of error of 2.0 and 1.0 percentage point, respectively^16^.

Data collection was performed using a single and standardized questionnaire, divided into blocks, which sought information about demographic, occupational, behavioral, and reproductive characteristics, as well as complications in this last pregnancy. We also collected information on housing conditions, possession of durable products, family crowding and socioeconomic level of the family.

The outcome, completion of adequate prenatal, was constituted from the criteria proposed by Takeda^4^ and Silveira et al.^5^ Takeda considered adequate when pregnant women began attending the appointments before the fifth month of pregnancy and attended six or more prenatal care appointments, while Silveira et al. classified as appropriate when pregnant women who attended six or more appointments began prenatal care before the fifth month of pregnancy and did two or more qualitative tests of urine, complete blood count and exams for the diagnosis of syphilis by the rapid test or the Venereal Disease Research Laboratory (VDRL).

Recent mothers were approached only once, in the maternity hospital, up to 48 hours after delivery. In the surveys of 2007, 2010 and 2013, printed and coded questionnaires were used by the interviewers and delivered at the headquarters of the project for further review, double typing, comparison and correction in EpiData 3.1^17^ and EpiInfo 6.04^16^ software. In 2016, the data were entered using tablets by the interviewers themselves via online Platform Web REDCap (Research Electronic Data Capture)^18^ with the server of the Universidade Federal do Rio Grande (FURG) at the address www.redcap.furg.br. The data entered were verified on the next day, when any inconsistencies were corrected.

The consistency and categorization of variables were analyzed in Stata 11^19^. Then the variables were assembled to assess the prenatal adequacy according to the criteria proposed by Takeda^4^ and Silveira et al.^5^ The proportions and the linear trend between 2007 and 2016 were assessed using chi-square test and trend tests, respectively. The confidence level employed was 95%. For quality control, 7% of the interviews were redone, i.e. every 15 questionnaires, one was chosen at random to be partially repeated. This mini questionnaire contained questions from all blocks of questions of the questionnaire. The answers were then compared. The agreement was assessed using the Kappa coefficient. In 2007, this coefficient ranged from 0.76 to 0.91; in 2010, from 0.63 to 0.78; in 2013, from 0.63 to 0.89; and in 2016, from 0.60 to 0.99. This shows satisfactory agreement^20^.

The research project was approved by the Research Ethics Committee in the Health Area of the Universidade Federal do Rio Grande (CEPAS/FURG) under numbers 23116.5369/6.58-12/2007, 23116.6258/9.64-117/2009, 23116.2623/67-007-2012 and 030/2015. Thus, all participants signed the informed consent form and received a copy of this document.

## RESULTS

In 2007, 2010, 2013 and 2016, the number of births was 10,669, and the mothers lived in the municipality of Rio Grande. Of this total, 10,331 (96.8%) mothers were interviewed.

Table 1 shows the main socioeconomic characteristics of recent mothers included in these surveys. Between 2007 and 2016, the rate of teenage mothers decreased 16% and labors among those of brown skin color increased 23%. The proportion of those who lived with a partner remained stable at around 84%. The schooling rate increased, especially among mothers who have graduated high school (≥ 12 years). The household income has improved, especially in categories of lower (up to 0.99 minimum wage) and higher (four minimum wages or more) income. The proportion of mothers who worked during pregnancy increased 23% over the period. The absence of prenatal appointments decreased and there was a gain, on average, of one prenatal appointment in the period.

**Table 1:** Main characteristics of recent mothers living in the municipality of Rio Grande, state of Rio Grande do Sul, who had children in 2007, 2010, 2013 or 2016.

Characteristic	Year of the perinatal study				p	Change (%) in the study period

	2007	2010	2013	2016		
Mother’s age (in years)					p < 0.001	
11 to 19	20.2	18.6	17.3	16.9		-16.3
20 to 24	28.1	26.8	26.3	26.1		-7.1
25 to 29	24.6	25.8	24.1	23.6		-4.1
30 or more	27.2	28.8	32.2	33.3		+22.4
Mean: (standard deviation)	25.6 (6.6)	25.9 (6.4)	26.3 (6.5)	26.5 (6.6)		-+0.9 (year)
Skin color					p < 0.001	
White	69.5	66.4	66.1	67.0		-3.6
Brown	18.3	20.6	22.3	22.6		+23.5
Black	12.2	9.9	11.7	10.3		−15.6
Mothers living with a partner	82.8	83.2	85.8	83.6	p = 0.02	+1.0
Schooling (years of study)					p < 0.001	
0 to 4	12.6	8.0	6.0	3.3		-73.8
5 to 8	36.1	37.2	33.6	33.4		-7.5
9 to 11	41.9	44.5	44.7	39.8		-5.0
12 or more	9.4	10.3	15.6	23.5		+150.0
Mean (standard deviation)	8.6 (3.5)	9.0 (3.2)	9.5 (3.3)	10.1 (3.6)		+1.5 (year)
Monthly household income in minimum wages (MW)					p < 0.001	
Up to 0.99	14.7	17.6	5.4	8.0		-45.6
1 to 1.99	32.5	33.5	28.5	29.1		-10.5
2 to 3.99	33.7	31.3	39.5	36.7		+8.9
4 or more	19.1	17.6	26.7	26.1		+36.6
Mean (standard deviation)	2.9 (3.2)	3.4 (10.5)	3.4 (3.5)	3.1 (3.1)		+0.3 (MW)
Mothers who work	37.4	42.8	43.6	45.9	p < 0.001	+22.7
Mothers who did not attend a single prenatal appointment	4.2	4.5	2.6	1.5	p < 0.001	-64.3
Mothers who began attending appointments in the first trimester of pregnancy	73.6	78.3	78.6	79.4	p < 0.001	+7.9
Mothers who attended six or more prenatal appointments	72.5	76.7	83.5	84.3	p < 0.001	+16.3
Mean (standard deviation)	7.4 (3.7)	7.7 (3.6)	8.3 (3.3)	8.2 (3.1)		+0.8 (appointment)

Total (n = 10,331)	2,557	2,395	2,685	2,694		

Table 2 shows the coverage of adequate prenatal care according to the household income between 2007 and 2016. According to Takeda, a significant increase occurred in the provision of adequate prenatal care, going from 69% to 81%, while for Silveira et al., this change was even higher, from 21% to 55% (p_trend_ < 0.001). This increase occurred in all income categories in both criteria used. After assessing the disparity between extreme categories of the same survey, a drastic reduction occurred, according to Takeda, falling from 37.7 p.p (or 74.5%) in 2007 to 28.3 p.p. (or 44.8%) in 2016, i.e. the disparity was reduced by one third over the period. Regarding the criterion proposed by Silveira et al., we observed the opposite: the disparity, which was 5.6 p.p. in 2007, increased to 14.8 p.p. in 2016, that is, an increase of 164% (14.8/5.6).

**Table 2:** Adequacy of prenatal care according to different criteria relating to the household income between recent mothers living in the municipality of Rio Grande, state of Rio Grande do Sul, 2007–2016.

Criterion/Monthly household income in minimum wages	Year of the perinatal survey				Change 2007–2016.		p for trend
	
	2007	2010	2013	2016	p.p.*	%	
			
	% (95%CI)	% (95%CI)	% (95%CI)	% (95%CI)			
Takeda	p < 0.001	p < 0.001	p < 0.001	p < 0.001			
Total	69.0 (67.2–70.8)	73.2 (71.5–75.0)	78.9 (77.4–80.5)	80.5 (79.1–82.1)	11.5	+16.7	p < 0.001
≤ 0.9	50.6 (45.9–56.0)	63.0 (58.4–67.7)	63.2 (55.2–71.2)	67.9 (61.6–74.2)	17.3	+34.2	p < 0.001
1 to 1.9	61.1 (57.8–64.4)	64.7 (61.4–68.0)	71.0 (67.8–74.2)	74.1 (71.0–77.2)	13.0	+21.3	p < 0.001
2 to 3.9	73.7 (70.7–76.6)	79.2 (76.2–82.1)	78.3 (75.8–80.8)	83.8 (81.6–86.1)	10.1	+13.7	p < 0.001
≥ 4	88.3 (85.4–91.2)	89.1 (86.1–92.1)	91.5 (89.4–93.5)	91.3 (90.0–93.7)	3.0	+3.4	p < 0.001
Difference between extreme income groups in the same survey							
p.p.*	37.7	26.1	28.3	23.4	-14.3	-61.1	
%	74.5	41.4	44.8	34.4	-40.1	-116.6	
Silveira et al.	p = 0.04	p < 0.001	p < 0.001	p < 0.001	33.4		
Total	21.3 (19.7–22.9)	44.0 (42.1–46.0)	57.0 (55.1–58.9)	54.7 (52.8–56.5)	28.0	+156.8	p < 0.001
≤ 0.9	19.4 (15.3–23.40)	34.8 (30.3–39.4)	36.8 (28.8–44.7)	47.4 (40.7–54.2)	28.9	+144.3	p = 0.036
1 to 1.9	20.2 (17.5–23.0)	37.2 (33.8–40.5)	50.3 (46.8–53.90)	49.1 (45.5–52.6)	36.1	+143.1	p < 0.001
2 to 3.9	21.1 (18.4–23.8)	49.9 (46.3–53.5)	57.2 (54.2–60.1)	57.2 (54.1–60.2)	37.2	+171.1	p < 0.001
≥ 4	25.0 (21.1–23.8)	55.9 (51.2–60.7)	68.0 (64.6–71.4)	62.2 (58.1–66.2)	37.2	+148.8	p < 0.001
Difference between extreme income groups in the same survey							
p.p.*	5.6	21.1	31.2	14.8	+9.2	+164.3	
%	28.9	60.6	84.8	31.2	+2.3	+7.9	

Total (n = 10,005)	2,449	2,288	2,614	2,654			

* percentage point.

## DISCUSSION

This study showed an increase in the coverage of adequate prenatal care in all income categories of both criteria used. It also showed the disparity reduction according to Takeda and increase regarding the criterion proposed by Silveira et al.

As to the assessment of the adequacy of prenatal care using the criterion by Takeda^4^, we verified the coverage of 81% in Caracol and Anísio de Abreu^21^, PI, in 2008; 59% in Santa Maria^22^, RS, in 2009; and 42% in João Pessoa^23^, PB, and 56% in Vitória^24^, ES, both in 2010. For the criteria proposed by Silveira et al.^5^, the adequacy rate found in Pelotas^25^, RS, was 37% in 2002, while in Palmas^26^, TO, it reached 69% in 2009. This study, however, analyzed only pregnant women treated in the FHS. In Rio Grande, the coverage rates for adequate prenatal care were 81% for Takeda and 55% for Silveira et al. The differences observed can be attributed to sample characteristics, the year of the study, socioeconomic level and the availability of health services in each of these locations.

Regarding the trend, for both criteria, we found that the provision of adequate prenatal care has increased consistently in the municipality, which is in agreement with the results observed in the cohorts of Pelotas^25^, São Luís and Ribeirão Preto.^9^ However, even for Takeda, undemanding criterion to achieve adequacy, there is room for improvement, while for Silveira et al., more robust criterion that requires two laboratory tests, the challenge is much bigger because almost half of them (45%) have not reached adequacy yet.

Despite the increase in the provision of adequate prenatal in all income categories in Rio Grande, we verified reduction and increase in disparity according to Takeda and Silveira et al., respectively. In the case of Takeda, the reduction was basically due to the expansion of the Family Health Strategy in the municipality, which went from 10 teams in 2007 to 36 teams in 2016, increasing the coverage of FHS from 38% to 57% over the period^27^. These teams were primarily installed in neglected areas or with insufficient capacity to meet the demand for prenatal care in these locals.

The fact that only the poor have a low number of appointments or do not receive prenatal care decreased the difference between extreme categories, reducing significantly the inequality in prenatal care, finding already evidenced in other studies^11^ ^,^ ^12^.

According to Silveira et al., despite the increase in prenatal coverage in all categories of income, the difference between extreme categories was higher in 2016 than in 2007, going from 5.6 p.p. in 2007 to 14.8 p.p. in 2016 (increase of 164%). This occurred because pregnant women with a better household income underwent tests of urine, VDRL and CBC in greater proportion than poor women, which explains the increase in both coverage and disparity. As to the fact that poor women get worse coverage, the care received by them is not satisfactory, either in quantity or in quality; at the same time, among the richest, the opposite occurs. This scenario was termed Inverse Care Law, qualification coined by JT. Hart in the 1970s^28^. More recently, other authors^29^ established a corollary to that law, stating that new technologies, such as more complex exams mentioned here, reach the rich first and only then the poor. Between 2007 and 2016 in Rio Grande, the coverage for these three tests among poor women increased from 91% to 94%, while it increased from 93% to 99% among the rich. These data may explain the increased disparity between these groups, since those with higher income achieved practically the universalization, while the poor had less evident improvement.

Finally, the increase in disparity regarding prenatal care found in Rio Grande is not in accordance with other studies on the subject^12^ ^,^ ^30^. A possible explanation is that other studies assessed only the number of appointments, but not the complementary exams as our study. If this analysis included only the criterion proposed by Takeda, restricted to the number of appointments and gestational age at initiation of prenatal care, the result would be similar.

When interpreting these results, one should consider the limitations inherent to the design used and the fact that they include questions since the planning of pregnancy until the immediate postpartum, that the questionnaire was applied a few hours after delivery, and that it took between 60 and 80 minutes. In addition, we included a technical question related to the kind of disparity assessed: our aim was to assess the difference between extremes, so we did not considerate the intermediate groups^31^. The assessment of intermediate groups requires more complex analysis of difficult interpretation, which would differ from our goal to denounce the increasing disparity between income extremes.

The provision of adequate prenatal care has been increasing consistently, but we must prioritize the provision of care for pregnant women with the worst household income. We suggest that other variables are included besides the number of appointments when assessing the adequacy of prenatal care, in order to provide a more in-depth analysis about the prenatal care, extremely relevant to the health care of mother and child.
